# Computational chemistry experiments performed directly on a blockchain virtual computer†

**DOI:** 10.1039/d0sc01523g

**Published:** 2020-04-21

**Authors:** Magnus W. D. Hanson-Heine, Alexander P. Ashmore

**Affiliations:** School of Chemistry, University of Nottingham University Park Nottingham NG7 2RD UK magnus.hansonheine@nottingham.ac.uk; School of Computing and Communications, The Open University Walton Hall, Kents Hill Milton Keynes MK7 6AA UK

## Abstract

Blockchain technology has had a substantial impact across multiple disciplines, creating new methods for storing and processing data with improved transparency, immutability, and reproducibility. These developments come at a time when the reproducibility of many scientific findings has been called into question, including computational studies. Here we present a computational chemistry simulation run directly on a blockchain virtual machine, using a harmonic potential to model the vibration of carbon monoxide. The results demonstrate for the first time that computational science calculations are feasible entirely within a blockchain environment and that they can be used to increase transparency and accessibility across the computational sciences.

## Abstract 2

Blockchain technology has had a substantial impact across multiple disciplines. This study proves that physical simulations can be performed entirely within a blockchain virtual machine. These simulations have improved reproducibility, provenance, and less potential for censorship.

## Graphical abstract

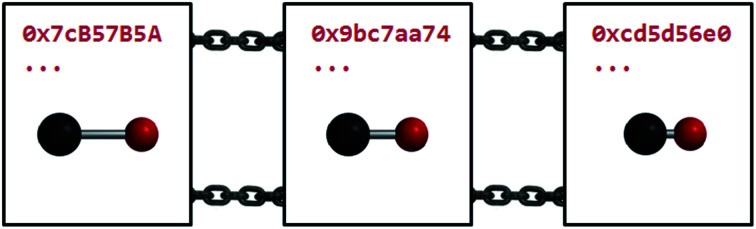

Distributed ledger technology has become an area of significant interest since the release of the first blockchain based crypto-currency, Bitcoin, in 2008.^1^ Since then blockchains have been used to increase transparency, immutability, and resistance to censorship in many areas outside of finance, including improving the reliability of medical trials,^2^ increasing energy efficiency,^3^ and allowing transparent and censorship resistant computation.^4^ The development of blockchain computation opens up the possibility of running computational science experiments. However, physical simulations have not previously been performed directly on blockchain virtual machines, and the first simulation of this kind is presented here.

Open public blockchains aim to create an electronic ledger with a provably tamperproof record of data that is available for anyone to review or add to in perpetuity without the possibility of censorship by a third party. This is carried out principally using a cryptographic hashing function that maps variable length input data to a fixed-length output called a hash. Any change to the input results in an unpredictable change to the hash, and blocks of newly appended data are required to contain a hash of the previous block so that revisions will invalidate the hash of the subsequent block and allow changes to be identified and removed automatically using a process known as “proof-of-work”.^1^ The details of how proof-of-work operates to maintain these properties have been discussed elsewhere.^1,4^

Several studies now indicate that a significant amount of the scientific literature cannot be replicated across a wide range of disciplines. In 2015 Aarts *et al.* examined reproducibility in the psychological scientific literature and found just over a third of the reproduced studies yielded statistically significant results compared to 97% of the original publications, with replication rates of roughly half in cognitive psychology and roughly a quarter in social psychology.^5^ A 2016 study of the economics literature by Camerer *et al.* found that *ca.* 39% of the sampled studies could not be replicated,^6^ and a 2018 study found that *ca.* 38% of social and behavioural science papers could not be replicated even when sampled exclusively from the journals *Nature* and *Science*.^7^ A 2008 meta-analysis by Fanelli also indicated that roughly 2% of the scientists surveyed had admitted to fabricating, falsifying or modifying data or results at least once, with roughly a third admitting to “other questionable research practices”,^8^ further emphasising the importance of replication.

Although reproducibility rates are expected to be higher in the computational sciences where simulations made up of a finite set of operations carried out on a deterministic computer allow for bit-for-bit replication, several publications have indicated that replication difficulties persist in fields as diverse as computational chemistry,^9^ harmonic analysis,^10^ and neuroscience.^11,12^ Computational replication is often hindered by a lack of access to the original output data, input files, software, hardware, and workflow, which can be difficult to maintain over extended periods of time.^13^ Reliably storing and accessing data can also be complicated by scientific censorship.^14^ Many rejections in peer-reviewed journals are due to quality control. However, there is evidence that some journal editors and referees can be hostile to work that challenges their current beliefs,^15–22^ which can delay or even prevent researchers from gaining access to peer-reviewed archiving services. In extreme cases, governments have also been known to remove or restrict access to the data provided by peer-reviewed journals.^23–25^

In 2015 the Ethereum network became the first instance of a blockchain acting as a virtual computer capable of performing general computation.^4^ Blockchain based computational science experiments can in principle solve many of the problems discussed for physical simulations by providing an effectively unchangeable record of the computational environment, including the exact piece of software used, a complete record of the associated computational steps, and open access for review and replication. However, computational science experiments using blockchains have not previously been performed. Software has not yet been developed to facilitate this kind of calculation, blockchains are not currently optimized for running these types of calculation, and the computational power of blockchains is still very limited compared to that of most conventional computers.

In order to prove that physical simulations can be performed using a blockchain, an atomistic molecular dynamics simulation was performed for the carbon monoxide molecule over a 40 fs time scale with a harmonic potential used to model the carbon–oxygen molecular bond. Software compatible with the Ethereum blockchain was written in the Solidity programming language in order to run a diatomic molecular dynamics trajectory with a variation of the velocity Verlet algorithm used to integrate Newton's equations of motion in atomic units.^26^ The simulation was executed for 400 time steps of 0.1 fs with an initial bond length of 120 pm. The model used an equilibrium bond length parameter of 112.8 pm and a force constant of 1855 N m^−1^, together with the masses of ^12^C and ^16^O assigned to the atoms, in order to model carbon monoxide. An equivalent simulation was written using the C# programming language, and executed on a local machine for comparison.

The molecular dynamics trajectories in Fig. 1 show that simulations of this kind can be performed entirely within a blockchain environment, and that doing so produces an identical output to local execution on a conventional machine within user specified precision of 1 × 10^−10^ a_0_. The details of this precision threshold, both algorithms, and their outputs can be found in the ESI.† The simulation that was carried out on the Ethereum network was also recorded on the blockchain in real time. The addition of both the code and simulation output into blocks of data on the blockchain happened as part of the process of running the simulation, and these entries can be used to track the provenance of the data for review and replication studies. The hashes and block numbers corresponding to these data on the Ethereum blockchain are included in the ESI† in addition to the discussion below, and can be used to both access the data and validate that the simulation record remains unchanged.

**Fig. 1:**
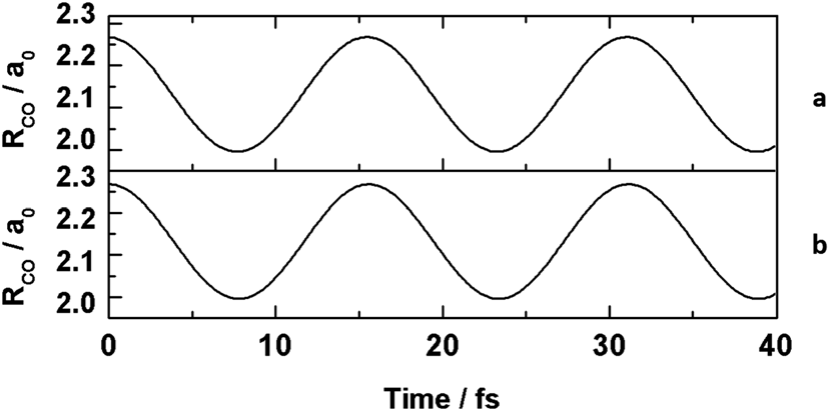
Molecular dynamics trajectories showing bond length variation over time for (a) the trajectory coded in C# and run on a local computer, and (b) the rescaled trajectory coded in Solidity and run on the Ethereum blockchain.

Timing when experiments occur can also be important for a number of reasons. When similar discoveries are made by independent researchers, the claims to the discoveries are often adjudicated based on when the specific observations or calculations were made, with famous examples including the controversy between Leibniz and Newton over who invented calculus. Knowing the order in which experiments were carried out is also useful when analyzing methods of hypothesis testing and conclusion formation that can differ depending on the order in which observations happen. An important property of many blockchains, including Ethereum, is therefore the creation of an internal chronology. New blocks are appended regularly, and the designed immutability of old data means that the position which calculations have in the blockchain acts as an automatic time stamp that can be used to verify the order in which they were performed. The computational complexity of solving the hashing function needed to append data is also commonly modified to give a regular time interval between blocks that can be used to approximate timings between different blockchains.^1,4^ In this case a preliminary trajectory of 10 time steps (1 fs) was run on the Ethereum blockchain prior to the main production run. The two trajectories have been recorded in blocks 9 360 161 and 9 360 178, respectively. This information provides an effective time stamp showing the order in which these simulations were performed. The output data and blockchain address of the preliminary simulation are also given in the ESI,† and the transaction and block details for both simulations are shown in Fig. 2.

**Fig. 2:**
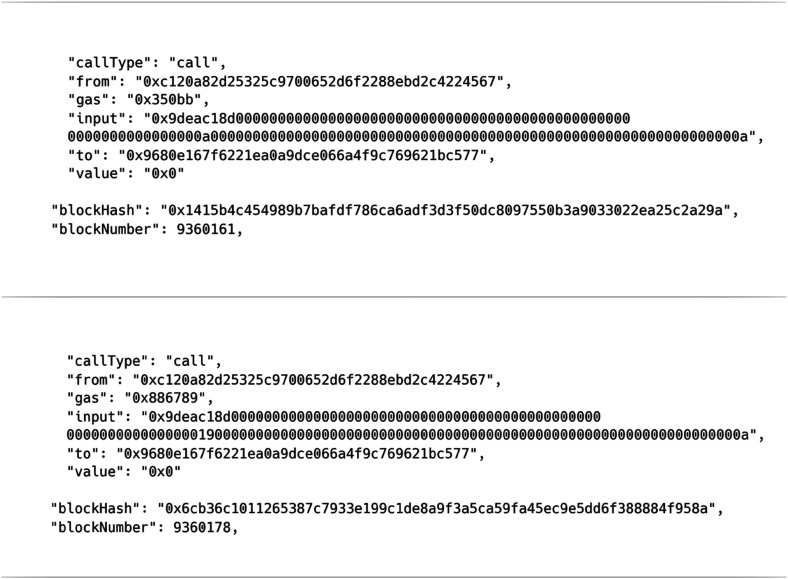
Transaction and block data for the 1 fs preliminary molecular dynamics trajectory (top panel) and the 40 fs production molecular dynamics trajectory (bottom panel).

The complexity and length of these simulations are currently limited by the capacity of the available blockchains. The Ethereum block for the production simulation was generated in *ca.* 11 s compared to a *ca.* 135 ms execution time for the C# simulation when executed on a standalone desktop machine running with a i7-4790k CPU and 32 GB of 1333 MHz DDR3 RAM. Each simulation was also recorded in a single block so as to avoid the need for manual interaction with the blockchain during their execution. While the exact computational power of the Ethereum network is variable, the network has a fixed limit to the maximum amount of computation that can be performed as part of generating a single block. This computational limit is measured in units known as gas, and is currently set at 10 000 000 gas at the time of writing. A more detailed description of the relationship between gas and computational operations can be found in the ESI† and associated resources. However, at this time we were unable to significantly increase the complexity of the simulation beyond the level reported. By comparison, large scale distributed computational science resources operating without distributed ledger technology can have significant computational throughput and storage requirements, with the well known example of the Folding@Home protein folding network reportedly calculating over 1 × 10^18^ operations per second earlier this year. Significant advancements in blockchain computer science and developments in combining “on-chain” and “off-chain” calculation data are therefore necessary before blockchain calculations can become a routine method for performing computational experiments.

## Conclusions

These results show that simulations of this kind are possible and that there can be significant benefits to using blockchains for computational science. The ability to run computational experiments with the properties outlined is expected to have an increasing impact across the computational sciences as the capacity of these blockchains continues to scale, and current plans to introduce blockchain database sharding to the Ethereum network are expected to produce a greater than 1000 fold increase in the computational throughput in the near future. Furthermore, running hybrid computational experiments that use blockchain based calculation and storage for certain parts of an experiment, and conventional off-chain computers for others, may allow some of these advantages to be introduced selectively at a significantly reduced computational cost.

## Conflicts of interest

The authors declare no competing financial interest.

## Supplementary Material

SC-011-D0SC01523G-s001
