# Supplementary material for: Computational chemistry experiments performed directly on a blockchain virtual computer
Source: Chem Sci. 2020 Apr 21;11(18):4644–7. doi: 10.1039/d0sc01523g (PMC8159212; doi:10.1039/d0sc01523g)
Supplement: SC-011-D0SC01523G-s001 [file SC-011-D0SC01523G-s001.pdf]

Electronic Supplementary Material (ESI) for Chemical Science.
This journal is © The Royal Society of Chemistry 2020

Computational chemistry experiments performed directly on a blockchain virtual computer

Magnus W. D. Hanson-Heine,[a]* and Alexander P. Ashmore[b]

[a]*School of Chemistry, University of Nottingham, University Park, Nottingham NG7 2RD, United Kingdom.*

[b]*School of Computing and Communications, The Open University, Walton Hall, Kents Hill, Milton Keynes MK7 6AA, United Kingdom.*

**magnus.hansonheine@nottingham.ac.uk*

Supplementary Details

Overview. The outputs from the two molecular dynamics simulations are reported in the Supplementary Information. The details of the interactions with the Ethereum blockchain where these simulations were performed and recorded have been reported using the block explorer tools on the website https://etherchain.org and are also given in the Supplementary Information. These data include the records of these simulations on the Ethereum blockchain, which should remain available for review and replication independent of this publication for as long as the blockchain persists. The results of these simulations are also reported as decimals in Tables S1 and S2 of the Supplementary Information, together with equivalent simulations performed using software written in the C# programming language and executed on a conventional local computer. The source codes for the C# and Solidity implementations of these simulations are also given and the compiled Solidity code is available on the Ethereum blockchain. The blockchain data can be found both in the Supplementary Information and on the blockchain in block number 9360156 with the address 0x9680e167f6221ea0a9dce066a4f9c769621bc577. Working knowledge of how blockchains operate, and how to execute software on the Ethereum network, as well as a downloadable copy of the Ethereum blockchain can be found at the time of writing on the websites https://ethereum.org/ and https://geth.ethereum.org/, and are necessary for review and replication of these data.

External Libraries. The mathematics used in these calculations makes use of decimal numbers. This is normally done in most programming languages through the use of doubles or floating point numbers, however, these are only precise up to a certain value. Two different computers generating slightly different results for the same double value can lead to slightly different simulation histories being recorded by different parts of the blockchain network. This can lead to the calculation being rejected as part of a consensus mechanism such as proof-of-work. Ethereum and similar blockchains that are capable of performing general computation have at the time of writing not yet implemented a method for calculating decimals with a fixed degree of precision, which would allow multiple machines to produce the same exact results from the same calculation. In this case, a third party library has been used in order to imitate floating point numbers using features that are currently available. In order to calculate these molecular dynamics trajectories, the ABDKMathQuad implements IEEE 754 standard fixed precision floating point numbers on the Ethereum blockchain. This library has been included in the code uploaded onto the blockchain as part of this work and can also be found at https://github.com/abdk-consulting/abdk-libraries-solidity/blob/master/ABDKMathQuad.md. Using this library, the results have been scaled by 10^p in order to provide accuracy up to p decimal places, where p is a variable supplied by the user when the code is called on the blockchain and chosen to be $p = 10$ for all of the calculations reported here. The output is then returned a set of integers by ABDKMathQuad and written as part of the simulation onto the blockchain as array of 256 bit integers in bytes displayed as a string of hexadecimal, and these have been divided by 10^p before being reported as decimals in Tables S1 and S2.

Computational Limit. The Ethereum network also has a limit to the maximum amount of computation that can be performed as part of generating a single block. This is measured in units known as gas, with the current limit being set at 10,000,000 gas at the time of writing. A more detailed description of the relationship between gas and computational operations can be found in the Ethereum paper “Ethereum: A Secure Decentralized Generalized Transaction Ledger EIP-150 Revision” by Dr. Gavin Wood on the website https://gavwood.com/paper.pdf at the time of writing.

Application Binary Interface. The procedure for calling a piece of software on the Ethereum blockchain varies depending on the type of software. If the code follows a standard format (such as the ERC20 token standard detailed in the Ethereum documentation) then most software for interacting with the code will already understand how to call it without the need for additional information. In the case of a new and therefore non-standard piece of software such as this, an Application Binary Interface (ABI) is needed to provide a description of the functions in the code.

Developers will usually provide this separately or it can be generated from the source code. The ABI for this contract is:


```
[
{
"constant": true,
"inputs": [
{
"internalType": "uint256",
"name": "steps",
"type": "uint256"
},
{
"internalType": "uint256",
"name": "precision",
"type": "uint256"
}
],
"name": "runMD",
"outputs": [
{
"internalType": "int256[]",
"name": "",
"type": "int256[]"
}
],
"payable": false,
"stateMutability": "pure",
"type": "function"
}
]
```

Preliminary Molecular Dynamics Trajectory Transaction Details

The details of the transaction and the corresponding blockchain output from the 1 fs molecular dynamics preliminary simulation on the Ethereum blockchain are presented here:

Block Number: 9360161
Hash: 0x13eb9c343f380334262706975c676aa5006ac2103b2132dfba0e1667c7dfddc6
Time Stamp: 01/26/2020 10:26:38 PM Greenwich Mean Time (GMT)
From: 0xC120A82d25325c9700652D6f2288EBD2C4224567
To: 0x9680E167F6221ea0A9dcE066A4f9c769621bC577
Fee: 0.00026 ETH (ca. $0.04)
Gas Price: 0.0000000011 ETH
Gas Used: 238,619

Trace [
{
"action": {
"callType": "call",
"from": "0xc120a82d25325c9700652d6f2288ebd2c4224567",
"gas": "0x350bb",
"input":
"0x9deac18d000000000000000000000000000000000000000000000000000000000000000a000
000000000000000000000000000000000000000000000000000000000000a",
"to": "0x9680e167f6221ea0a9dce066a4f9c769621bc577",
"value": "0x0"
},
"blockHash":
"0x1415b4c454989b7bafdf786ca6adf3d3f50dc8097550b3a9033022ea25c2a29a",
"blockNumber": 9360161,
"result": {
"gasUsed": "0x350bb",
"output":
"0x00000000000000000000000000000000000000000000000000000000000000200000000000000
0000000000000000000000000000000000000000000000000000a000000000000000000000000
0000000000000000000000000000000547924e860000000000000000000000000000000000000
0000000000000000005475fb0cd000000000000000000000000000000000000000000000000000
00005470b6c020000000000000000000000000000000000000000000000000000000054695a339
0000000000000000000000000000000000000000000000000000000545fe877c00000000000000
000000000000000000000000000000000000000005454657b000000000000000000000000000
00000000000000000000000000005446d60820000000000000000000000000000000000000000
00000000000000054373fc44000000000000000000000000000000000000000000000000000000
05425a92c70000000000000000000000000000000000000000000000000000000541219930"
},
"subtraces": 0,
"traceAddress": [],
"transactionHash":
"0x13eb9c343f380334262706975c676aa5006ac2103b2132dfba0e1667c7dfddc6",
"transactionPosition": 43,
"type": "call"
}
]

Production Molecular Dynamics Trajectory Transaction Details
The details of the transaction and the corresponding blockchain output from the 40 fs molecular dynamics production simulation on the Ethereum blockchain are presented here:

Block Number: 9360178
Hash: 0x36b510f3bdb2fa67a0d4f749899ab8630b54000ee5a211172d699d750f94f94f
Time Stamp: 01/26/2020 10:30:26 PM Greenwich Mean Time (GMT)
From: 0xC120A82d25325c9700652D6f2288EBD2C4224567
To: 0x9680E167F6221ea0A9dcE066A4f9c769621bC577
Fee: 0.00986 ETH (ca. $1.66)
Gas Price: 0.0000000011 ETH
Gas Used: 8,960,757

Trace [
{
"action": {
"callType": "call",
"from": "0xc120a82d25325c9700652d6f2288ebd2c4224567",
"gas": "0x886789",
"input":
"0x9deac18d0000000000000000000000000000000000000000000000000000000000190000
000000000000000000000000000000000000000000000000000000000000000a",
"to": "0x9680e167f6221ea0a9dce066a4f9c769621bc577",
"value": "0x0"
},
"blockHash":
"0x6cb36c1011265387c7933e199c1de8a9f3a5ca59fa45ec9e5dd6f388884f958a",
"blockNumber": 9360178,
"result": {
"gasUsed": "0x886789",
"output":
"0x000000000000000000000000000000000000000000000000000000000000002000000000000
0000000000000000000000000000000000000000000019000000000000000000000000
000000000000000000000000000000547924e8600000000000000000000000000000000000
0000000000000000005475fb0cd000000000000000000000000000000000000000000000
00005470b6c020000000000000000000000000000000000000000000000000000054695a339
000000000000000000000000000000000000000000000000000000545fe877c000000000000000
000000000000000000000000000000000000005454657b00000000000000000000000000000
000000000000000000000000000005446d6082000000000000000000000000000000000000000
00000000000000054373fc44000000000000000000000000000000000000000000000000000
05425a92c7000000000000000000000000000000000000000000000000000000541219930000
00000000000000000000000000000000000000000000000053fc991c900000000000000000
000000000000000000000000000000000053e530bc800000000000000000000000000000
00000000000000000000000053cbea3150000000000000000000000000000000000000000000
0000000000053b0d000800000000000000000000000000000000000000000000000000000005
393ed72700000000000000000000000000000000000000000000000000000053754e8d300000
00000000000000000000000000000000000000000000000053550010100000000000000000000
000000000000000000000000000000000053330f6dd000000000000000000000000000000000
0000000000000000000000000530f8ac760000000000000000000000000000000000000000000
00000000052ea80e5e00000000000000000000000000000000000000000000000000000052c4
01346000000000000000000000000000000000000000000000000000000529c1bb9a00000000
000000000000000000000000000000000000000000005272e1116000000000000000000000
0000000000000000000000000000000052486265300000000000000000000000000000000000
0000000000000000000000521cb165c000000000000000000000000000000000000000000000
0000000051efe04320000000000000000000000000000000000000000000000000000000051c201
a560000000000000000000000000000000000000000000000000000000519328a4b0000000000

0000000000000000000000000000000000000000000000000516368c1900000000000000000000000
000000000000000000000000000000000005132d5dc80000000000000000000000000000000000000
0000000000000000005101842e0000000000000000000000000000000000000000000000000000000
00000050cf883dc00000000000000000000000000000000000000000000000000000000000509cf6da
a00000000000000000000000000000000000000000000000000000000005069e51160000000000000
0000000000000000000000000000000000000000000000503668248000000000000000000000000000
00000000000000000000000000000050029582e000000000000000000000000000000000000000000
000000000000000004fce82bf200000000000000000000000000000000000000000000000000000000
0004f9a4586b000000000000000000000000000000000000000000000000000000000004f65f3987000
000000000000000000000000000000000000000000000000000000004f31a2bc200000000000000000
0000000000000000000000000000000000000000004efd68b8f0000000000000000000000000000000
0000000000000000000000004ec95b4c8000000000000000000000000000000000000000000000000
000000000004e959021f000000000000000000000000000000000000000000000000000000000004e6
21cc8c000000000000000000000000000000000000000000000000000000000004e2f16ac000000000
0000000000000000000000000000000000000000000000000004dfc930920000000000000000000000
0000000000000000000000000000000004dcaa6e75000000000000000000000000000000000000000
000000000000000000004d99670eb0000000000000000000000000000000000000000000000000000
0000004d68e7ffb0000000000000000000000000000000000000000000000000000000000004d393de
a7000000000000000000000000000000000000000000000000000000004d0a7ca670000000000000
000000000000000000000000000000000000000000000004cdcb7aa400000000000000000000000000
0000000000000000000000000000004cb00203700000000000000000000000000000000000000000000
000000000000000004c846e4ea0000000000000000000000000000000000000000000000000000000
000004c5a0eafa000000000000000000000000000000000000000000000000000000000004c30f4ca3
00000000000000000000000000000000000000000000000000000000004c0931ba500000000000000
00000000000000000000000000000000000000000000004be2d60d6000000000000000000000000000
000000000000000000000000000004bbdf1bb200000000000000000000000000000000000000000000
0000000000000004b9a941f10000000000000000000000000000000000000000000000000000000000
04b78cbf20000000000000000000000000000000000000000000000000000000000004b58a743f0000
00000000000000000000000000000000000000000000000000000004b3a337680000000000000000
000000000000000000000000000000000000000004b1d7d36c0000000000000000000000000000000
0000000000000000000000004b029078800000000000000000000000000000000000000000000000
0000000000004ae978712000000000000000000000000000000000000000000000000000000000004
ad23f92c000000000000000000000000000000000000000000000000000000000004abcef883000000
000000000000000000000000000000000000000000000000000004aa9913090000000000000000000
0000000000000000000000000000000000000004a982c9c1000000000000000000000000000000000
000000000000000000000004a88c9083000000000000000000000000000000000000000000000000
00000000004a7b6cdcd000000000000000000000000000000000000000000000000000000000004a7
01da9c000000000000000000000000000000000000000000000000000000004a66e02440000000000
0000000000000000000000000000000000000000000000000004a5fb825000000000000000000000000
00000000000000000000000000000000004a5aa8a6a00000000000000000000000000000000000000
0000000000000000000004a57b3c47000000000000000000000000000000000000000000000000000
000000004a56dab9700000000000000000000000000000000000000000000000000000000004a581
de0100000000000000000000000000000000000000000000000000000000004a5b7cb1c000000000
000000000000000000000000000000000000000000000000004a60f5c7500000000000000000000000
000000000000000000000000000000004a6886d990000000000000000000000000000000000000000
00000000000000000004a722cc2100000000000000000000000000000000000000000000000000000
0000004a7de37cb000000000000000000000000000000000000000000000000000000000004a8ba62
92000000000000000000000000000000000000000000000000000000000004a9b6f0ce000000000000
000000000000000000000000000000000000000000000004aad3795f00000000000000000000000000
000000000000000000000000000004ac0f85d100000000000000000000000000000000000000000000
0000000000000004ad6a92940000000000000000000000000000000000000000000000000000000000
004aee40f30000000000000000000000000000000000000000000000000000000000004b07b5e80000
00000000000000000000000000000000000000000000000000000000004b22fd6f7000000000000000
00000000000000000000000000000000000000000004b400c2e300000000000000000000000000000
0000000000000000000000004b5ed60ba00000000000000000000000000000000000000000000000
0000000000004b7f4e36b000000000000000000000000000000000000000000000000000000000004b

a1672b4000000000000000000000000000000000000000000000000000000004bc512b78000000
00000000000000000000000000000000000000000000000004bea420230000000000000000000
0000000000000000000000000000000000004c10e590b00000000000000000000000000000000
000000000000000000000004c38ed4d4000000000000000000000000000000000000000000000
00000000004c62488df00000000000000000000000000000000000000000000000000000004c8c
e61b9000000000000000000000000000000000000000000000000000000004cb8b438b00000000
0000000000000000000000000000000000000000000000004ce5a0a9000000000000000000000
000000000000000000000000000000000004d1398b930000000000000000000000000000000000
0000000000000000000004d428946600000000000000000000000000000000000000000000000
000000004d725ec65000000000000000000000000000000000000000000000000000000004da30
54f7000000000000000000000000000000000000000000000000000000004dd468a130000000000
00000000000000000000000000000000000000000000004e06742c70000000000000000000000
00000000000000000000000000000004e39131be000000000000000000000000000000000000
0000000000000000004e6c305d000000000000000000000000000000000000000000000000000
0000004e9fb6a8c00000000000000000000000000000000000000000000000000000004ed3908c
20000000000000000000000000000000000000000000000000000000004f07a87190000000000000
00000000000000000000000000000000000000004f3be8a99000000000000000000000000000
00000000000000000000000004f703b73e0000000000000000000000000000000000000000
00000000000004fa48b0870000000000000000000000000000000000000000000000000000000
4fd8c1a0c000000000000000000000000000000000000000000000000000000500cc980600000
000000000000000000000000000000000000000000000000050408cfea0000000000000000000
000000000000000000000000000000000005073f68f2000000000000000000000000000000000
000000000000000000000050a6f0cb000000000000000000000000000000000000000000000000
00000000050d96679a000000000000000000000000000000000000000000000000000000000510b4
299b000000000000000000000000000000000000000000000000000000000513c7069c000000000
000000000000000000000000000000000000000000000516cdb70d0000000000000000000000
000000000000000000000000000000000519c6f87200000000000000000000000000000000000
00000000000000000051cb18de60000000000000000000000000000000000000000000000000
00000051f8c409f000000000000000000000000000000000000000000000000000000000052255e06f
000000000000000000000000000000000000000000000000000005250d4447000000000000000
0000000000000000000000000000000000000000527b14aaa00000000000000000000000000
0000000000000000000000000000052a40da3000000000000000000000000000000000000000
000000000000000052cbae1f1000000000000000000000000000000000000000000000000000
0052f1e5a000000000000000000000000000000000000000000000000000000005316a43d1000
000000000000000000000000000000000000000000000000000005339daaa700000000000000000
00000000000000000000000000000000000000535b7a3f9000000000000000000000000000000
0000000000000000000000000537b74fd600000000000000000000000000000000000000000000
000000000005399bd93e000000000000000000000000000000000000000000000000000000053
b64767c000000000000000000000000000000000000000000000000000000053d10697b000000
000000000000000000000000000000000000000000000000053e9f00180000000000000000000000
000000000000000000000000000000000005400f94640000000000000000000000000000000000
00000000000000000000541618cf10000000000000000000000000000000000000000000000000
0000000542945d0e00000000000000000000000000000000000000000000000000000000543a78
5030000000000000000000000000000000000000000000000000000000005449a92450000000000
000000000000000000000000000000000000000000005456d1fa7000000000000000000000000
0000000000000000000000000000005461ed58200000000000000000000000000000000000000
000000000000000000546af69dc0000000000000000000000000000000000000000000000000
00005471ea08600000000000000000000000000000000000000000000000000000005476c4b34
000000000000000000000000000000000000000000000000000000547984991000000000000000
00000000000000000000000000000000000000547a2894d0000000000000000000000000000
000000000000000000000000000005478b0624000000000000000000000000000000000000000
000000000000000054751c9df000000000000000000000000000000000000000000000000000000
00546f6ec51000000000000000000000000000000000000000000000000000000005467a934f000
000000000000000000000000000000000000000000000000000545dcf29b00000000000000000
00000000000000000000000000000000000005451e4bd40000000000000000000000000000000
0000000000000000000000005443eee570000000000000000000000000000000000000000000000

0000000000005433f371f000000000000000000000000000000000000000000000000000000054
21f909f0000000000000000000000000000000000000000000000000000000540e072970000000
00000000000000000000000000000000000000000000000053f8261dd000000000000000000000
000000000000000000000000000000000053e05f02b00000000000000000000000000000000000
0000000000000000000053c6bbbe0000000000000000000000000000000000000000000000000
000000053ab46fbf0000000000000000000000000000000000000000000000000000000538e0c2
a70000000000000000000000000000000000000000000000000000000536f17746000000000000
0000000000000000000000000000000000000000000534e75bcd0000000000000000000000000
000000000000000000000000000000532c34991000000000000000000000000000000000000000
000000000000000005308624bb000000000000000000000000000000000000000000000000000
000052e30dbe00000000000000000000000000000000000000000000000000000052bc467a5
000000000000000000000000000000000000000000000000000000052941ca510000000000000
000000000000000000000000000000000000000000526aa0f67000000000000000000000000000
0000000000000000000000000000523fe4b3200000000000000000000000000000000000000000
000000000000005213f9a570000000000000000000000000000000000000000000000000000000
51e6f21590000000000000000000000000000000000000000000000000000000051b8e0c2500000
000000000000000000000000000000000000000000000000005189d8d8e000000000000000000
00000000000000000000000000000000000005159eded50000000000000000000000000000000
000000000000000000000000512933f22000000000000000000000000000000000000000000000
000000000050f7bf2ff000000000000000000000000000000000000000000000000000000050c5a
43d300000000000000000000000000000000000000000000000000000005092f7f580000000000
000000000000000000000000000000000000000000000505fcf71000000000000000000000000
0000000000000000000000000000000502c3ffb700000000000000000000000000000000000000
000000000000000004ff85f0ba00000000000000000000000000000000000000000000000000000
004fc4423a000000000000000000000000000000000000000000000000000000004f8fff38300000
000000000000000000000000000000000000000000000000004f5babc7a0000000000000000000
0000000000000000000000000000000000004f275db08000000000000000000000000000000000
00000000000000000000004ef32ab8e00000000000000000000000000000000000000000000000
000000004ebf289b900000000000000000000000000000000000000000000000000000004e8b6c
fee00000000000000000000000000000000000000000000000000000004e580d6be00000000000
000000000000000000000000000000000000000000004e251f4560000000000000000000000000
0000000000000000000000000000004df2b7bed000000000000000000000000000000000000000
00000000000000004dc0ebd3c0000000000000000000000000000000000000000000000000000
0004d8fd03ef00000000000000000000000000000000000000000000000000000004d5f79719000
00000000000000000000000000000000000000000000000000004d2ffb8b100000000000000000
000000000000000000000000000000000000004d016a509000000000000000000000000000000
00000000000000000000000004cd3d924a0000000000000000000000000000000000000000000
0000000000004ca75aff300000000000000000000000000000000000000000000000000000004c
7c0265d00000000000000000000000000000000000000000000000000000004c51e163d000000
00000000000000000000000000000000000000000000000004c290982b0000000000000000000
0000000000000000000000000000000000004c018bc3200000000000000000000000000000000
000000000000000000000004bdb78958000000000000000000000000000000000000000000000
00000000004bb6dfd380000000000000000000000000000000000000000000000000000000004b93
d0b900000000000000000000000000000000000000000000000000000004b7259de500000000
000000000000000000000000000000000000000000000004b528931a000000000000000000000
00000000000000000000000000000000004b346bf1500000000000000000000000000000000000
000000000000000000004b180ea6b000000000000000000000000000000000000000000000000
00000004afd7d2070000000000000000000000000000000000000000000000000000000004ae4c26
de00000000000000000000000000000000000000000000000000000004acde8da700000000000
000000000000000000000000000000000000000000004ab8f9e920000000000000000000000000
0000000000000000000000000000004aa5fe50c000000000000000000000000000000000000000
00000000000000004a94fdf8500000000000000000000000000000000000000000000000000000
004a85fff390000000000000000000000000000000000000000000000000000004a790a8040000
0000000000000000000000000000000000000000000000000004a6e2303500000000000000000
000000000000000000000000000000000000004a654e06d000000000000000000000000000000
00000000000000000000000004a5e8f38000000000000000000000000000000000000000000000

000000000004a59e965a00000000000000000000000000000000000000000000000000000004a
575e7f000000000000000000000000000000000000000000000000000000004a56ef9330000000
0000000000000000000000000000000000000000000000004a589cd0400000000000000000000
000000000000000000000000000000000004a5c65838000000000000000000000000000000000
00000000000000000000004a624819c0000000000000000000000000000000000000000000000
0000000004a6a421fe00000000000000000000000000000000000000000000000000000004a745
043b00000000000000000000000000000000000000000000000000000004a806e55b000000000
00000000000000000000000000000000000000000000004a8e974aa0000000000000000000000
0000000000000000000000000000000004a9ec53d600000000000000000000000000000000000
000000000000000000004ab0f171c0000000000000000000000000000000000000000000000000
0000004ac51457300000000000000000000000000000000000000000000000000000004adb258
bc00000000000000000000000000000000000000000000000000000004af31bdfd000000000000
00000000000000000000000000000000000000000004b0ced59c0000000000000000000000000
0000000000000000000000000000004b288f3a4000000000000000000000000000000000000000
00000000000000004b45f600700000000000000000000000000000000000000000000000000000
004b65156f300000000000000000000000000000000000000000000000000000004b85e091c000
00000000000000000000000000000000000000000000000000004ba849c170000000000000000
0000000000000000000000000000000000000004bcc42ab300000000000000000000000000000
000000000000000000000000004bf1bc5560000000000000000000000000000000000000000000
0000000000004c18a72680000000000000000000000000000000000000000000000000000004
c40f2eb100000000000000000000000000000000000000000000000000000004c6a8edd0000000
00000000000000000000000000000000000000000000000004c9569aa00000000000000000000
0000000000000000000000000000000000004cc1717b200000000000000000000000000000000
000000000000000000000004cee93fc30000000000000000000000000000000000000000000000
0000000004d1cbe6340000000000000000000000000000000000000000000000000000000004d4b
dd78900000000000000000000000000000000000000000000000000000004d7bdd9e700000000
000000000000000000000000000000000000000000000004dacaad9a000000000000000000000
00000000000000000000000000000000004dde30d960000000000000000000000000000000000
0000000000000000000004e105b00200000000000000000000000000000000000000000000000
000000004e43146bf00000000000000000000000000000000000000000000000000000004e7647
ff300000000000000000000000000000000000000000000000000000004ea9e069800000000000
000000000000000000000000000000000000000000004eddc8308000000000000000000000000
00000000000000000000000000000004f11e9b8a00000000000000000000000000000000000000
000000000000000004f462f4e80000000000000000000000000000000000000000000000000000
0004f7a832f900000000000000000000000000000000000000000000000000000004faecf936000
00000000000000000000000000000000000000000000000000004fe2feb4b0000000000000000
000000000000000000000000000000000000005016fada400000000000000000000000000000000
000000000000000000000000504aae60300000000000000000000000000000000000000000000
0000000000050507e03c0d00000000000000000000000000000000000000000000000000000050
b0e59da000000000000000000000000000000000000000000000000000000050e33ec83000000
00000000000000000000000000000000000000000000000005114fa4b000000000000000000000
0000000000000000000000000000000000051460372400000000000000000000000000000000000
0000000000000000000000517645d430000000000000000000000000000000000000000000000
00000000051a5ad5a0000000000000000000000000000000000000000000000000000000051d4
2647e000000000000000000000000000000000000000000000000000000052019d45600000000
00000000000000000000000000000000000000000000000522dff656000000000000000000000
00000000000000000000000000000000052593a2e100000000000000000000000000000000000
0000000000000000000052833ba09000000000000000000000000000000000000000000000000
000000052abf240700000000000000000000000000000000000000000000000000000000052d34d1
af000000000000000000000000000000000000000000000000000000052f93bce1000000000000
0000000000000000000000000000000000000000000531dae8f700000000000000000000000000
0000000000000000000000000000053409632f0000000000000000000000000000000000000000
0000000000000005361e430b00000000000000000000000000000000000000000000000000000
0053818aaba0000000000000000000000000000000000000000000000000000000539f7c76d000
000000000000000000000000000000000000000000000000000053bbad1b10000000000000000
00000000000000000000000000000000000000053d610dc400000000000000000000000000000

0000000000000000000000000053ee9cbe2000000000000000000000000000000000000000000
000000000000054054688c0000000000000000000000000000000000000000000000000000000
541a04cd10000000000000000000000000000000000000000000000000000000542ccee870000
000000000000000000000000000000000000000000000000000543d9d08800000000000000000
00000000000000000000000000000000000000544c682e7000000000000000000000000000000
000000000000000000000000054592a31b0000000000000000000000000000000000000000000
0000000000005463ddc2a00000000000000000000000000000000000000000000000000000005
46c7e6ce000000000000000000000000000000000000000000000000000000054730899000000
000000000000000000000000000000000000000000000000005477798e1000000000000000000
00000000000000000000000000000000000005479cf72d00000000000000000000000000000000
00000000000000000000000547a094e6000000000000000000000000000000000000000000000
0000000000547827089000000000000000000000000000000000000000000000000000000547
4296a30000000000000000000000000000000000000000000000000000000546e121c90000000
0000000000000000000000000000000000000000000000005465e3a8c00000000000000000000
00000000000000000000000000000000000545ba176b000000000000000000000000000000000
0000000000000000000000544f4fcb900000000000000000000000000000000000000000000000
000000005440f3c820000000000000000000000000000000000000000000000000000000543093
66c0000000000000000000000000000000000000000000000000000000541e3578a0000000000
0000000000000000000000000000000000000000000005409e1a3000000000000000000000000
0000000000000000000000000000000053f3a05c30000000000000000000000000000000000000
00000000000000000053db7ae7d00000000000000000000000000000000000000000000000000
0000053c17b532000000000000000000000000000000000000000000000000000000053a5ac70
b0000000000000000000000000000000000000000000000000000000538819d40000000000000
00000000000000000000000000000000000000000005368cfcc800000000000000000000000000
000000000000000000000000000005347db60b000000000000000000000000000000000000000
000000000000000053254a4870000000000000000000000000000000000000000000000000000
00053012ae7600000000000000000000000000000000000000000000000000000052db8c4730
00000000000000000000000000000000000000000000000000000052b47e10c00000000000000
00000000000000000000000000000000000000000528c10863000000000000000000000000000
00000000000000000000000000005262547bd0000000000000000000000000000000000000000
00000000000000052375b5130000000000000000000000000000000000000000000000000000
00520b36e9e000000000000000000000000000000000000000000000000000000051ddf9a60000
000000000000000000000000000000000000000000000000000051afb65aa00000000000000000
0000000000000000000000000000000000000051808049f0000000000000000000000000000000
00000000000000000000000051506b1b100000000000000000000000000000000000000000000
00000000000511f8ad220000000000000000000000000000000000000000000000000000000050e
df3c7c000000000000000000000000000000000000000000000000000000050bbbaa0900000000
000000000000000000000000000000000000000000000005088f444d0000000000000000000000
0000000000000000000000000000000005055b5d7600000000000000000000000000000000000
00000000000000000000502214ad5000000000000000000000000000000000000000000000000
00000004fee2644b00000000000000000000000000000000000000000000000000000004fba003
b900000000000000000000000000000000000000000000000000000004f85b8476000000000000
00000000000000000000000000000000000000000004f51642bb00000000000000000000000000
000000000000000000000000000004f1d19b0e0000000000000000000000000000000000000000
0000000000000004ee8ee9b900000000000000000000000000000000000000000000000000000
004eb4f8a340000000000000000000000000000000000000000000000000000004e814d694000
00000000000000000000000000000000000000000000000000004e4e026fc00000000000000000
000000000000000000000000000000000000004e1b2d10f0000000000000000000000000000000
0000000000000000000000004de8e276200000000000000000000000000000000000000000000
000000000004db7378ea00000000000000000000000000000000000000000000000000000004d
86410780000000000000000000000000000000000000000000000000000004d561342a000000
00000000000000000000000000000000000000000000000004d26c24e40000000000000000000
0000000000000000000000000000000000004cf861dcc000000000000000000000000000000000
00000000000000000000004ccb053c80000000000000000000000000000000000000000000000
0000000004c9ebf4f90000000000000000000000000000000000000000000000000000004c73a
283f00000000000000000000000000000000000000000000000000000004c49c0cc30000000000

0000000000000000000000000000000000000000000004c212b97600000000000000000000000
000000000000000000000000000000004bf9f3ca80000000000000000000000000000000000000
0000000000000000004bd429b8e00000000000000000000000000000000000000000000000000
000004bafdd1d90000000000000000000000000000000000000000000000000000000004b8d1d14f
00000000000000000000000000000000000000000000000000000004b6bf816500000000000000
000000000000000000000000000000000000000004b4c7bedc000000000000000000000000000
00000000000000000000000000004b2eb5b680000000000000000000000000000000000000000
0000000000000004b12b1d5a00000000000000000000000000000000000000000000000000000
004af87bf4a00000000000000000000000000000000000000000000000000000004ae01efcb0000
0000000000000000000000000000000000000000000000000004ac9a512200000000000000000
000000000000000000000000000000000000004ab51790300000000000000000000000000000
00000000000000000000000004aa27f05200000000000000000000000000000000000000000000
000000000004a91e32eb00000000000000000000000000000000000000000000000000000004a
834af6c00000000000000000000000000000000000000000000000000000004a76bc70d0000000
0000000000000000000000000000000000000000000000004a6c3cd6d00000000000000000000
000000000000000000000000000000000004a63d087b000000000000000000000000000000000
00000000000000000000004a5d7b04f00000000000000000000000000000000000000000000000
000000004a593ef1c00000000000000000000000000000000000000000000000000000004a571e
1140000000000000000000000000000000000000000000000000000004a57194640000000000
0000000000000000000000000000000000000000000004a593092e00000000000000000000000
000000000000000000000000000000004a5d6318300000000000000000000000000000000000
00000000000000000004a63af16f00000000000000000000000000000000000000000000000000
000004a6c11f010000000000000000000000000000000000000000000000000000000004a768825e
00000000000000000000000000000000000000000000000000000004a830d5d70000000000000
0000000000000000000000000000000000000000004a919c60900000000000000000000000000
000000000000000000000000000004aa22f1fb0000000000000000000000000000000000000000
0000000000000004ab4beb4c00000000000000000000000000000000000000000000000000000
004ac94365a"
    },
    "subtraces": 0,
    "traceAddress": [],
    "transactionHash":
"0x36b510f3bdb2fa67a0d4f749899ab8630b54000ee5a211172d699d750f94f94f",
    "transactionPosition": 10,
    "type": "call"
  }
]

Molecular Dynamics C# Source Code

```
using System;
using System.Collections.Generic;

namespace DiatomicMD.CommandLine
{
    class Program
    {
        static void Main(string[] args)
        {
            var proc = new DiatomicMD();
            var res = proc.RunMD();
            foreach (var result in res) Console.WriteLine(result);
            Console.ReadKey();
        }
    }

    /// <summary>
    /// Initializes a diatomic molecular dynamics trajectory for the carbon monoxide molecule run
with a variation of the velocity Verlet algorithm used to integrate Newton's equations of motion in
atomic units.
    /// </summary>
    public class DiatomicMD
    {
        public DiatomicMD()
        {
            R1 = 2.26767135; // !initial bond length in a0(120.0 pm)
            TMax = 400; // !total number of time steps
            R2 = 0;
            V1 = 0;
            V2 = 0;
        }

        public const double Re = 2.1316; // !equilibrium bond length in a0(112.8 pm)
        public const double K = 1.1915; // !reciprocal force constant in a_0 ^ 2 / Eh(19.02 N / cm)
        public const double M1 = 21875; // !mass of atom one in m_e(Carbon)
        public const double M2 = 29156.9457; // !mass of atom two in m_e(Oxygen)
        public const double DT = 4.13; // !0.1 fs time step in hbar / Eh
        public static double dTbyM1x2 => DT / (M1 * 2);
        public static double dTbyM2x2 => DT / (M2 * 2);
        public static double dTdTbyM1x2 => (DT * DT) / (M1 * 2);
        public static double dTdTbyM2x2 => (DT * DT) / (M2 * 2);

        public double Rmag { get; set; }
        public double R { get; set; }
        public double R1 { get; set; }
        public double R2 { get; set; }
        public double V1 { get; set; }
        public double V2 { get; set; }
        public double F { get; set; }
        public double FNew { get; set; }
        public double T { get; set; }
        public double TMax { get; set; }

        /// <summary>
```

```
        /// Executes a diatomic molecular dynamics trajectory for the carbon monoxide molecule run with a variation of the velocity Verlet algorithm used to integrate Newton's equations of motion in atomic units.
        /// </summary>
        /// <returns></returns> A list of doubles representing the carbon monoxide bond length in atomic units.
        public List<double> RunMD()
        {
            var res = new List<double>();
            T = 0;
            while (T < TMax)
            {
                R = R1 - R2;
                Rmag = Math.Abs(R);
                F = -K * (Rmag - Re) * R / Rmag;
                R1 = R1 + DT * V1 + dTdTbyM1x2 * F;
                R2 = R2 + DT * V2 - dTdTbyM2x2 * F;

                R = R1 - R2;
                Rmag = Math.Abs(R);

                FNew = -K * (Rmag - Re) * R / Rmag;

                V1 = V1 + dTbyM1x2 * (FNew + F);
                V2 = V2 - dTbyM2x2 * (FNew + F);

                res.Add(Rmag);
                T++;
            }
            return res;
        }
    }
}
```

Molecular Dynamics Solidity Source Code

The Solidity source code for the molecular dynamics simulation that was used to run these simulations on the Ethereum blockchain is given here:

```
pragma solidity ^0.5.16;
import "github.com/abdk-consulting/abdk-libraries-solidity/ABDKMathQuad.sol";
/**
```


* Copyright (c) 2019, ABDK Consulting

* All rights reserved.

* Redistribution and use in source and binary forms, with or without modification, are permitted provided that the following conditions are met:

* Redistributions of source code must retain the above copyright notice, this list of conditions and the following disclaimer.
* Redistributions in binary form must reproduce the above copyright notice, this list of conditions and the following disclaimer in the documentation and/or other materials provided with the distribution.
* All advertising materials mentioning features or use of this software must display the following acknowledgement: This product includes software developed by ABDK Consulting.
* Neither the name of ABDK Consulting nor the names of its contributors may be used to endorse or promote products derived from this software without specific prior written permission.
* THIS SOFTWARE IS PROVIDED BY ABDK CONSULTING "AS IS" AND ANY EXPRESS OR IMPLIED WARRANTIES, INCLUDING, BUT NOT LIMITED TO, THE IMPLIED WARRANTIES OF MERCHANTABILITY AND FITNESS FOR A PARTICULAR PURPOSE ARE DISCLAIMED. IN NO EVENT SHALL ABDK CONSULTING BE LIABLE FOR ANY DIRECT, INDIRECT, INCIDENTAL, SPECIAL, EXEMPLARY, OR CONSEQUENTIAL DAMAGES (INCLUDING, BUT NOT LIMITED TO, PROCUREMENT OF SUBSTITUTE GOODS OR SERVICES; LOSS OF USE, DATA, OR PROFITS; OR BUSINESS INTERRUPTION) HOWEVER CAUSED AND ON ANY THEORY OF LIABILITY, WHETHER IN CONTRACT, STRICT LIABILITY, OR TORT (INCLUDING NEGLIGENCE OR OTHERWISE) ARISING IN ANY WAY OUT OF THE USE OF THIS SOFTWARE, EVEN IF ADVISED OF THE POSSIBILITY OF SUCH DAMAGE.


```
**/

/// @title Diatomic Molecular Dynamics
/// @notice A diatomic molecular dynamics trajectory for the carbon monoxide molecule run with a variation of the velocity Verlet algorithm used to integrate Newton's equations of motion in atomic units.
/// @author Alex Ashmore <kezyma@yahoo.co.uk>, Magnus Hanson-Heine <magnus.hansonheine@gmail.com>
contract DiatomicMD {

    bytes16 constant Re = 0x400010d844d013a92a305532617c1bda;
// 2.1316      - equilibrium bond length in a_0 (112.8 pm)
    bytes16 constant K = 0x3fff310624dd2f1a9fbe76c8b4395810;
// 1.1915      - force constant in Eh/a_0^2 (18.55 N/cm)
    bytes16 constant M1 = 0x400d55cc000000000000000000000000;
// 21875       - mass of atom one in m_e (Carbon)
    bytes16 constant M2 = 0x400dc793c86594af4f0d844d013a92a3;
// 29156.9457  - mass of atom two in m_e (Oxygen)
    bytes16 constant dT = 0x40010851eb851eb851eb851eb851eb85;
// 4.13        - 0.1 fs time step in hbar/E_h
    bytes16 constant dTdTbyM1x2 = 0x3ff398cf77d58230a4ec10bd70161b26;
// ((dT*dT)/(M1*2))
    bytes16 constant dTdTbyM2x2 = 0x3ff332b5c5317be96318c8a728b2194e;
```

```
// ((dT*dT)/(M2*2))
    bytes16 constant dTbyM1x2 = 0x3ff18bf13a6a5f196b0b10d53a599821;
// (dT/(M1*2))
    bytes16 constant dTbyM2x2 = 0x3ff1290e444148485119dcfda2c40c1e;
// (dT/(M2*2))

    /// @notice Initializes a molecular dynamics trajectory
    /// @param steps Number of steps in time in units of 0.1 femtosecond
    /// @param precision Number of decimal places to include in output
    function runMD(uint256 steps, uint precision) public returns (int256[] memory) {

        bytes16 r1 = 0x400022430e0729bfecea7985fddb6291; // 2.26767135  - initial bond length in
a_0 (120.0 pm)
        bytes16 r2 = 0x00000000000000000000000000000000; // 0
        bytes16 v1 = 0x00000000000000000000000000000000; // 0
        bytes16 v2 = 0x00000000000000000000000000000000; // 0

        bytes16 r;
        bytes16 rMag;
        bytes16 f;
        bytes16 fNew;

        uint256 t = 0;

        uint ten = 10;
        uint multiplier = ten**precision;
        bytes16 convertedMultiplier = ABDKMathQuad.fromUInt(multiplier);
        int256[] memory results = new int256[](steps);

        while (t < steps) {
            r = ABDKMathQuad.sub(r1,r2);
            rMag = ABDKMathQuad.abs(r);

            f =
ABDKMathQuad.div(ABDKMathQuad.mul(ABDKMathQuad.mul(ABDKMathQuad.neg(K),ABDKM
athQuad.sub(rMag,Re)),r),rMag);

            r1 =
ABDKMathQuad.add(ABDKMathQuad.add(r1,ABDKMathQuad.mul(dT,v1)),ABDKMathQuad.mul(
dTdTbyM1x2,f));
            r2 = ABDKMathQuad.sub(ABDKMathQuad.add(r2,ABDKMathQuad.mul(dT,v2)),
ABDKMathQuad.mul(dTdTbyM2x2,f));

            r = ABDKMathQuad.sub(r1,r2);
            rMag = ABDKMathQuad.abs(r);

            fNew =
ABDKMathQuad.div(ABDKMathQuad.mul(ABDKMathQuad.mul(ABDKMathQuad.neg(K),ABDKM
athQuad.sub(rMag,Re)),r),rMag);

            v1 =
ABDKMathQuad.add(v1,ABDKMathQuad.mul(dTbyM1x2,ABDKMathQuad.add(fNew,f)));
            v2 =
ABDKMathQuad.sub(v2,ABDKMathQuad.mul(dTbyM2x2,ABDKMathQuad.add(fNew,f)));

            results[t] = ABDKMathQuad.toInt(ABDKMathQuad.mul(rMag, convertedMultiplier));
```

```
            t = t + 1;
        }
        return results;
    }
}
```

Molecular Dynamics Solidity Source Code Upload Transaction Details

The details of the transaction in which the Solidity source code for the molecular dynamics simulation was uploaded onto the Ethereum blockchain are presented here:

Block Number: 9360156
Hash: 0x1c98dbb671dbe76b7cb4188d7585296e5adbe0f64fe8144546b4a82081089152
Time Stamp: 01/26/2020 10:25:44 PM Greenwich Mean Time (GMT)
From: 0xC120A82d25325c9700652D6f2288EBD2C4224567
To: 0x9680E167F6221ea0A9dcE066A4f9c769621bC577
Fee: 0.00091 ETH (ca. $0.15)
Gas Price: 0.0000000011 ETH
Gas Used: 825,151

Trace [
{
"action": {
"from": "0xc120a82d25325c9700652d6f2288ebd2c4224567",
"gas": "0xaeb1b",
"init":
"0x608060405234801561001057600080fd5b50610df6806100206000396000f3fe6080604052348
01561001057600080fd5b506004361061002b5760003560e01c80639deac18d14610030575b600
080fd5b61005360048036036040811015610046576000080fd5b50803590602001356100a3565b6
04080516020808252835181830152835191928392908301918581019102808383600005b838110
1561008f5781810151838201526020016100775650505050905001925050506040518091039O
f35b60606f400022430e0729bfecea7985fddb629160801b600080808080808080600a8b810a826
100d1826102dd565b905060608f6040519080825280602002602001820160405280156100ff5781
602001602082028038833901905050b5090505b8f8510156102c8576101158d8d610343565b9850
6101208961035c565b9750610175610l6f6101696101476f03fff310624dd2f1a9fbe76c8b4395816
0841b61036b565b6101648c6f2000086c226809d495182a9930be0ded60811b610343565b61037
4565b8b610374565b896105f2565b96506101bd6101a08e61019b6f40010851eb851eb851eb851
eb851eb8560801b8f610374565b61087d565b61019b6f1ff9cc67bbeac1185276085eb80b0d9360
811b8a610374565b9c506102056101e38d61019b6f40010851eb851eb851eb851eb851eb856080
1b8e610374565b6102006f1ff9995ae298bdf4b18c645394590ca760811b8a610374565b6103435
65b9b506102118d8d610343565b985061021c8961035c565b97506102436101 6f6101696101476
f03fff310624dd2f1a9fbe76c8b43958160841b61036b565b955061026a8b61019b6f3ff18bf13a6a5f
196b0b10d53a59982160801b6101648a8c61087d565b9a506102918a6102006f1ff894872220a42
4288cee7ed162060f60811b6101648a8c61087d565b99506102a56102a08984610374565b610c6
b565b8186815181106102b157fe5b6020026020010181815250508460010194506101035 65b9c5
05050505050505050505050505b92915050565b6000816102ec5750600061033e565b81600061
02f882610d1e565b9050607081101561031157806070038290 1b9150610324565b607081111561
03245760708103829 01c91505b613fff0160701b6001600160701b0391909116176 0801b90505b9
19050565b600061035583600160ff1b841861087d565b9392505050565b600160801b600160ff1b
031690565b600160ff1b1890565b6000617fff60f084811c8216919084901c8116908214156 10424
5780617fff14156103f25760016001 60801b031985811690851614156103bf575050506001 60ff1b8
11682186102d7565b600160ff1b6001600160801b03198686181614156103e2575050508181176
102d7565b5061ffff60ef1b91506102d79050565b600160801b600160ff1b038416610413575061ffff
60ef1b91506102d79050565b505050600160ff1b811682186102d7565b80617fff1415610460 5760
0160801b600160ff1b03851661044f575061ffff60ef1b91506102d79050565b505050600160ff1b82
1681186102d7565b6001600160701b03608086901c168261047c576001925061048 3565b60016
0701b175b6001600160701b03608086901c168261049f57600192506104a6565b600160701b175
b90810290816104d457600160ff1b87871816610 4c35760006104c9565b600160ff1b5b94505050
50506102d7565b9282019260006001 60e11b8310156105085760 0160e01b83101561050057610
4fb83610d1e565b610503565b60e05b61050b565b60e15b905061407081860110156105265760 0
094506000925 06105c3565b6140e0818601101561056957614070851015610 54b578461407003
83901c9250610560565b6140708511156105605761407085038390 1b92505b600094506105c35
65b61c0dd8186011115610583576 17fff94506000925 06105c3565b607081111561059a57607081

0383901c92506105ad565b60708110156105ad578060700383901b92505b6001600160701b038
31692506140df8186010394505b82607086901b888a186001607f1b60801b1660801c600160016
0801b0316171760801b9550505050505506102d7565b6000617fff60f084811c8216919084901c81
16908214156106275780617fff1415610413575061ffff60ef1b91506102d79050565b80617fff14156
1066b576dffffffffffffffffffffffffffff60801b84161561065a575061ffff60ef1b91506102d79050565b50505
0808218600160ff1b166102d7565b600160801b600160ff1b0384166106b557600160801b600160f
f1b03851661069d575061ffff60ef1b91506102d79050565b5050508082186001600ff1b16617fff60f0
1b176102d7565b6001600160701b03608085901c16816106d15760019150610 6d8565b6001607
01b175b6001600160701b03608087901c16836107175780156107125760006106fc82610d1e565
b6001955060e2039384016071190193919091 1b90505b610721565b600160701b1760721b5b81
818161072a57fe5b04905080610746576000160ff1b87871816610 4c35760006104c9565b6001606
c1b81101561075457fe5b6000600160731b821015610794576001 60721b821015610789576001 6
0711b821015610781576070610784565b60715b61078c565b60725b60ff1661079d565b61079d8
2610d1e565b905083614071018186011156107bb57617fff9450600091506108 4e565b83818601
613ffc0110156107d65760009450600091506108 4e565b83818601613f8c011015610823578385 6
13ffc011115610801578385613ffc010382901b915061081a565b8385613ffc01101561081a57613f
fc8585030382901c91505b600094506108 4e565b6070811115610836576070810382901c91505b
6001600160701b0382169150838186016 13f8d010394505b81607086901b888a186001607f1b60
801b1660801c6001600160801b0316171760801b9550505050505506102d7565b6000617fff60f08
4811c8216919084901c81169082141561 08cb5780617fff14156108c157600160 0160801b031985
8116908516141561 03e257849250505 06102d7565b8492505050 6102d7565b80617fff14156108d
f578392505050 6102d7565b6001607f1b608086901c90811015906001600160701b0316836109 0
5576001935061090c565b600160701b175b6001607f1b608087901c9081101590600160 0160701
b03168461093257600194506109 39565b600160701b175b826109695760016001600160801b031988
16600160ff1b1461095957876109 5c565b60005b9650505050505050506102d7565b806109895760
01600160801b0319891660 0160ff1b146109595788610 95c565b848603841515831515141561 0a
ac5760708113156109b05789975050505050505050506102d7565b60008113156109cf5780826000
8212156109c657fe5b901c9150610a09565b606f198112156109e857888975050505050505050 61
02d7565b600081121561 0a095780600003846000821215610a0157fe5b901c935085965 05b9281
0192600160711b8410610a2457600196870196939093 1c925b86617fff1415610a565784610a3e5
7617fff60f01b610a48565b6001600160f01b03195b97505050505050505 06102d7565b600160701
b841015610a6b5760009650610a78565b6001600160701b03841693505b83607088901b86610a
8a576000610a90565b6001607f1b5b6001600160801b0316171760801b97505050505050505 061
02d7565b6000811315610ac757600184901b9350600187039650610ade565b60008112156 10ad
e57600182901b9150600186039650 5b6070811315610af05760019150610b53565b60018113156
10b1857600181036001830360008212156 10b0c57fe5b901c6001019150610b53565b606f19811
215610b2b5760019350610b53565b600019811215610b5357600181600003036001850360008 2
1215610b4b57fe5b901c60010193505b818410610b645781840393506 10b6d565b838203935082
94505b83610b8357506000965 06102d7955050505050505 0565b6000610b8e85610d1e565b90508
06071141561 0bb457600185901c6001600160701b0316945060018801975 0610c03565b607081
1015610bf65760708190038089111561 0be3578086901b6001600160701b031695508089039850
610bf0565b600098600019019590951b945b50610c03565b6001600160701b0385169450 5b8761
7fff1415610c365785610c1d57617fff60f01b610c27565b6001600160f01b03195b9850505050505
05050506102d7565b84607089901b87610c48576000610c4e565b6001607f1b5b6001600160801
b0316171760801b98505050505050505050506102d7565b6000617fff60f083901c166140fe8111156
10c8557600080fd5b613fff811015610c995760009150506103 3e565b600160701b600160016070
1b03608085901c161761406f821015610cc35761406f8290031c610cd5565b61406f821115610cd
55761406e1982011b5b6001607f1b608085901c10610d0157600160ff1b81111561 0cf557600080f
d5b6000039150610 33e9050565b6001600160ff1b038111156 10d1557600080fd5b915061033e90
50565b600080821161 0d2c57600080fd5b6000600160801b8310610d4157608092831c92015b68
0100000000000000008310610d5957604092831c92015b6401000000008310610d6d576020928
31c92015b620100008310610d7f57601092831c92015b6101008310610d9057600892831c92015
b60108310610da057600492831c92015b60048310610db057600292831c92015b600283106102
d757600101929150 5056fea265627a7a72315820d72739475f1b26be10735eab39ccaf43992cf00f
66b7e7fbdb4a3cad038216a264736f6c63430005100032",
"value": "0x0"

},
"blockHash":
"0x82e933a530bf6299df1522de1c4a2cca609d2d2ec13cd04a07fced2b2062bb78",
"blockNumber": 9360156,
"result": {
"address": "0x9680e167f6221ea0a9dce066a4f9c769621bc577",
"code":
"0x608060405234801561001057600080fd5b506004361061002b5760003560e01c80639deac18
d14610030575b600080fd5b61005360048036036040811015610046576000080fd5b50803590602
001356100a3565b60408051602080825283518183015283519192839290830191858101910280
838360005b8381101561008f578181015183820152602001610077565b505050509050019250 50
5060405180910390f35b60606f400022430e0729bfecea7985fddb629160801b600080808080808
080600a8b810a826100d1826102dd565b905060608f604051908082528060200260200182016 04
05280156100ff578160200160208202803883390190505b5090505b8f8510156102c8576101158d
8d610343565b9850610120896103 5c565b97506101756101 6f610169610147 6f03fff310624dd2f1
a9fbe76c8b43958160841b61036b565b6101648c6f2000086c226809d495182a9930be0ded6081
1b610343565b610374565b8b610374565b896105f2565b96506101bd6101a08e61019b6f400108
51eb851eb851eb851eb851eb8560801b8f610374565b61087d565b61019b6f1ff9cc67bbeac1185
276085eb80b0d9360811b8a610374565b9c506102056101e38d61019b6f40010851eb851eb851e
b851eb851eb8560801b8e610374565b6102006f1ff9995ae298bdf4b18c645394590ca760811b8a
610374565b610343565b9b506102118d8d610343565b985061021c8961035c565b97506102436
1016f6101696101476f03fff310624dd2f1a9fbe76c8b43958160841b61036b565b955061026a8b61
019b6f3ff18bf13a6a5f196b0b10d53a59982160801b6101648a8c61087d565b9a506102918a6102
006f1ff894872220a424288cee7ed162060f60811b6101648a8c61087d565b99506102a56102a08
984610374565b610c6b565b8186815181106102b157fe5b602002602001018181525050846001 0
19450610103565b9c5050505050505050505050505b92915050565b6000816102ec57506000
61033e565b8160006102f882610d1e565b90506070811015610311578060700382901b91506103
24565b60708111156103245760708103 82901c91505b613fff0160701b6001600160701b0391909
1161760801b90505b919050565b60006103558 3600160ff1b841861087d565b93925050505 65b6
00160801b600160ff1b031690565b600160ff1b1890565b6000617fff60f084811c8216919084901c
81169082141561042457806 17fff14156103f2576001600160801b0319858116908516141561 03bf
57505050600160ff1b811682186102d7565b600160ff1b6001600160801b031986861816141561 0
3e25750505081811761 02d7565b5061ffff60ef1b91506102d79050565b600160801b600160ff1b03
8416610413575061ffff60ef1b91506102d79050565b505050600160ff1b811682186102d7565b806
17fff141561046057600160801b600160ff1b03851661044f575061ffff60ef1b91506102d79050565b
505050600160ff1b821681186102d7565b6001600160701b03608086901c168261047c57600192
50610483565b600160701b175b6001600160701b03608086901c168261049f57600192506104a6
565b600160701b175b90810290816104d457600160ff1b878718166104c35760006104c9565b60
0160ff1b5b9450505050506102d7565b928201926000600160e11b831015610508576001 60e01b
831015610500576104fb83610d1e565b610503565b60e05b61050b565b60e15b905061407081 86
01101561052657600094506000925061 05c3565b6140e08186011015610569576140708510156
1054b578461407003839 01c9250610560565b6140708511156105605761407085038 3901b9250
5b600094506105c3565b61c0dd8186011115610583576 17fff94506000925061 05c3565b6070811
11561059a5760708103 83901c92506105ad565b6070811015610 5ad578060700383901b92505b
6001600160701b038316925061 40df8186010394505b82607086901b888a186001607f1b60801b
1660801c6001600160801b0316171760801b955050505050506102d7565b6000617fff60f084811
c8216919084901c811690821415610627578061 7fff1415610413575061ffff60ef1b91506102d790
50565b80617fff141561066b576dffffffffffffffffffffffffffff60801b84161561065a575061ffff60ef1b91506
102d79050565b505050808218600160ff1b166102d7565b600160801b600160ff1b0384166106b5
57600160801b600160ff1b03851661069d575061ffff60ef1b91506102d79050565b505050808218 6
00160ff1b16617fff60f01b176102d7565b6001600160701b03608085901c16816106d157600191 5
06106d8565b600160701b175b6001600160701b03608087901c16836107175780156107125760
006106fc82610d1e565b6001955060e20393840160711901939190911b90505b610721565b6001
60701b1760721b5b818181610 72a57fe5b04905080610746576001 60ff1b878718166104c357600
06104c9565b6001606c1b81101561075457fe5b6000600160731b82101561079457600160721b8
21015610789576001 60711b82101561078157607061 0784565b60715b61078c565b60725b60ff1

661079d565b61079d82610d1e565b905083614071018186011156107bb57617fff945060009150
61084e565b83818601613ffc0110156107d6576000945060009150610 84e565b83818601613f8c0
110156108235783856 13ffc01111561080157838561 3ffc010382901b915061081a565b8385613ff
c01101561081a57613ffc8585030382901c91505b6000945061084e565b607081111561083657 6
070810382901c91505b6001600160701b03821691508381860 1613f8d010394505b81607086901
b888a186001607f1b60801b1660801c6001600160801b0316171760801b95505050505050506102d
7565b6000617fff60f084811c8216919084901c8116908214156108cb5780617fff14156108c15760
01600160801b031985811690851614156103e2578492505050506102d7565b84925050506102d75
65b80617fff14156108df5783925050506102d7565b6001607f1b608086901c90811015906001600
160701b031683610905576001935061090c565b600160701b175b6001607f1b608087901c90811
015906001600160701b031684610932576001945061093956 5b600160701b175b826109695760
01600160801b0319881660 0160ff1b14610959578761095c565b60005b965050505050505050 6102
d7565b80610989576001600160801b0319891660 0160ff1b14610959578861095c565b84860384
15158315151415610aac576070811315610 9b057899750505050505050505 06102d7565b6000811
3156109cf57808260008212156109c657fe5b901c9150610a09565b606f198112156109e8578897
50505050505050506102d7565b6000811215610a095780600003846000821215610a0157fe5b90
1c93508596505b92810192600160711b8410610a245760019687019693909 31c925b86617fff141
5610a565784610a3e57617fff60f01b610a48565b6001600160f01b03195b975050505050505050 6
102d7565b600160701b841015610a6b5760009650610a78565b6001600160701b03841693505b
83607088901b86610a8a576000610a90565b6001607f1b5b6001600160801b0316171760801b97
5050505050505050 6102d7565b6000811315610ac757600184901b93506001870 39650610ade5
65b6000811215610ade57600182901b9150600186039 6505b6070811315610af05760019150610
b53565b6001811315610b1857600181036001830360008212 15610b0c57fe5b901c60010191506
10b53565b606f19811215610b2b5760019350610b53565b60001981121 5610b535760018160000
3036001850360008212 15610b4b57fe5b901c60010193505b818410610b6457818403935 0610b6
d565b83820393508294505b83610b835750600096506102d79550505050505050565b6000610b8e
85610d1e565b905080607114156 10bb457600185901c6001600160701b0316945060018801975
0610c03565b607081101561 0bf657607081900380891115610be3578086901b6001600160701b0
3169550808903985 0610bf0565b6000986000190195909 51b945b50610c03565b6001600160701
b03851694505b87617fff1415610c365785610c1d57617fff60f01b610c27565b6001600160f01b03
195b9850505050505050505 06102d7565b84607089901b87610c48576000610c4e565b6001607f
1b5b6001600160801b0316171760801b985050505050505050505 06102d7565b6000617fff60f0839
01c166140fe811115610c8557600080fd5b613fff811015610c995760009150506103 3e565b60016
0701b6001600160701b03608085901c161761406f821015610cc35761406f8290031c610cd5565b
61406f821115610cd55761406e1982011b5b6001607f1b608085901c10610d0157600160ff1b811
115610cf557600080fd5b6000039150610 33e9050565b6001600160ff1b03811115610d15576000
80fd5b915061033e9050565b6000808211610d2c57600080fd5b6000600160801b8310610d4157
608092831c92015b68010000000000000000 8310610d5957604092831c92015b6401000000008
310610d6d57602092831c92015b62010000 8310610d7f57601092831c92015b61010083 10610d9
057600892831c92015b60108310610da057600492831c92015b60048310610db057600292831c
92015b600283106102d7576001019291505056fea265627a7a72315820d72739475f1b26be1073
5eab39ccaf43992cf00f66b7e7fbdb4a3cad038216a264736f6c63430005100032",
      "gasUsed": "0xaeb1b"
    },
    "subtraces": 0,
    "traceAddress": [],
    "transactionHash":
"0x1c98dbb671dbe76b7cb4188d7585296e5adbe0f64fe8144546b4a82081089152",
    "transactionPosition": 56,
    "type": "create"
  }
]

Table S1. Calculated bond lengths (a_0) and simulation times (fs) for the preliminary run molecular dynamics trajectory of 10 steps when run on Ethereum network using the Solidity language and on a local computer using the C# language.

Time	Bond Length (Solidity)	Bond Length (C#)
0.1	2.2675607174	2.2675607175
0.2	2.2672289997	2.2672289997
0.3	2.2666767362	2.2666767362
0.4	2.2659048249	2.2659048250
0.5	2.2649145212	2.2649145212
0.6	2.2637074352	2.2637074352
0.7	2.2622855298	2.2622855298
0.8	2.2606511172	2.2606511173
0.9	2.2588068551	2.2588068552
1.0	2.2567557424	2.2567557425

Column 1 shows the elapsed simulation time in fs. Columns 2-3 show the calculated carbon-oxygen bond lengths (in a_0) predicted by the Solidity and C# implementations of the trajectory, respectively.

Table S2. Ccalculated bond lengths (a_0) and simulation times (fs) for the production run molecular dynamics trajectory of 400 steps when run on Ethereum network using the Solidity language and on a local computer using the C# language.

Time	Bond Length (Solidity)	Bond Length (C#)
0.1	2.2675607174	2.2675607175
0.2	2.2672289997	2.2672289997
0.3	2.2666767362	2.2666767362
0.4	2.2659048249	2.2659048250
0.5	2.2649145212	2.2649145212
0.6	2.2637074352	2.2637074352
0.7	2.2622855298	2.2622855298
0.8	2.2606511172	2.2606511173
0.9	2.2588068551	2.2588068552
1.0	2.2567557424	2.2567557425
1.1	2.2545011145	2.2545011146
1.2	2.2520466376	2.2520466376
1.3	2.2493963029	2.2493963029
1.4	2.2465544200	2.2465544201
1.5	2.2435256103	2.2435256103
1.6	2.2403147987	2.2403147988
1.7	2.2369272065	2.2369272066
1.8	2.2333683421	2.2333683422
1.9	2.2296439926	2.2296439927
2.0	2.2257602142	2.2257602142
2.1	2.2217233222	2.2217233222
2.2	2.2175398810	2.2175398811
2.3	2.2132166934	2.2132166934
2.4	2.2087607891	2.2087607892
2.5	2.2041794140	2.2041794141
2.6	2.1994800178	2.1994800179
2.7	2.1946702422	2.1946702423
2.8	2.1897579083	2.1897579084
2.9	2.1847510041	2.1847510041
3.0	2.1796576712	2.1796576713
3.1	2.1744861920	2.1744861920
3.2	2.1692449756	2.1692449757
3.3	2.1639425450	2.1639425450
3.4	2.1585875222	2.1585875223
3.5	2.1531886152	2.1531886152
3.6	2.1477546030	2.1477546030
3.7	2.1422943218	2.1422943218
3.8	2.1368166507	2.1368166507
3.9	2.1313304967	2.1313304968
4.0	2.1258447810	2.1258447811
4.1	2.1203684239	2.1203684240
4.2	2.1149103304	2.1149103304
4.3	2.1094793759	2.1094793759
4.4	2.1040843916	2.1040843917
4.5	2.0987341504	2.0987341504
4.6	2.0934373522	2.0934373522
4.7	2.0882026101	2.0882026101
4.8	2.0830384363	2.0830384364
4.9	2.0779532283	2.0779532284

5.0	2.0729552551	2.0729552552
5.1	2.0680526439	2.0680526440
5.2	2.0632533668	2.0632533669
5.3	2.0585652279	2.0585652280
5.4	2.0539958506	2.0539958506
5.5	2.0495526650	2.0495526650
5.6	2.0452428963	2.0452428963
5.7	2.0410735525	2.0410735526
5.8	2.0370514134	2.0370514135
5.9	2.0331830194	2.0331830195
6.0	2.0294746609	2.0294746609
6.1	2.0259323680	2.0259323680
6.2	2.0225619007	2.0225619008
6.3	2.0193687400	2.0193687400
6.4	2.0163580780	2.0163580780
6.5	2.0135348104	2.0135348105
6.6	2.0109035282	2.0109035282
6.7	2.0084685100	2.0084685101
6.8	2.0062337155	2.0062337155
6.9	2.0042027785	2.0042027786
7.0	2.0023790017	2.0023790018
7.1	2.0007653507	2.0007653507
7.2	1.9993644493	1.9993644494
7.3	1.9981785756	1.9981785757
7.4	1.9972096580	1.9972096581
7.5	1.9964592720	1.9964592721
7.6	1.9959286378	1.9959286379
7.7	1.9956186183	1.9956186183
7.8	1.9955297175	1.9955297176
7.9	1.9956620801	1.9956620801
8.0	1.9960154908	1.9960154908
8.1	1.9965893749	1.9965893750
8.2	1.9973827993	1.9973827993
8.3	1.9983944737	1.9983944737
8.4	1.9996227531	1.9996227531
8.5	2.0010656402	2.0010656402
8.6	2.0027207886	2.0027207887
8.7	2.0045855071	2.0045855071
8.8	2.0066567633	2.0066567633
8.9	2.0089311892	2.0089311892
9.0	2.0114050864	2.0114050864
9.1	2.0140744320	2.0140744321
9.2	2.0169348855	2.0169348856
9.3	2.0199817955	2.0199817956
9.4	2.0232102074	2.0232102075
9.5	2.0266148715	2.0266148716
9.6	2.0301902516	2.0301902516
9.7	2.0339305336	2.0339305336
9.8	2.0378296355	2.0378296356
9.9	2.0418812171	2.0418812171
10.0	2.0460786900	2.0460786900
10.1	2.0504152287	2.0504152288
10.2	2.0548837817	2.0548837818

10.3	2.0594770827	2.0594770827
10.4	2.0641876624	2.0641876625
10.5	2.0690078611	2.0690078611
10.6	2.0739298406	2.0739298406
10.7	2.0789455973	2.0789455973
10.8	2.0840469751	2.0840469752
10.9	2.0892256787	2.0892256788
11.0	2.0944732871	2.0944732871
11.1	2.0997812670	2.0997812670
11.2	2.1051409872	2.1051409873
11.3	2.1105437324	2.1105437324
11.4	2.1159807170	2.1159807171
11.5	2.1214431001	2.1214431002
11.6	2.1269219993	2.1269219994
11.7	2.1324085054	2.1324085054
11.8	2.1378936967	2.1378936968
11.9	2.1433686540	2.1433686540
12.0	2.1488244742	2.1488244743
12.1	2.1542522858	2.1542522859
12.2	2.1596432626	2.1596432627
12.3	2.1649886384	2.1649886384
12.4	2.1702797210	2.1702797211
12.5	2.1755079067	2.1755079068
12.6	2.1806646940	2.1806646940
12.7	2.1857416973	2.1857416973
12.8	2.1907306610	2.1907306611
12.9	2.1956234726	2.1956234726
13.0	2.2004121759	2.2004121759
13.1	2.2050889839	2.2050889840
13.2	2.2096462919	2.2096462919
13.3	2.2140766890	2.2140766891
13.4	2.2183729712	2.2183729712
13.5	2.2225281521	2.2225281522
13.6	2.2265354752	2.2265354752
13.7	2.2303884241	2.2303884241
13.8	2.2340807335	2.2340807335
13.9	2.2376063993	2.2376063994
14.0	2.2409596886	2.2409596887
14.1	2.2441351486	2.2441351486
14.2	2.2471276156	2.2471276156
14.3	2.2499322235	2.2499322236
14.4	2.2525444120	2.2525444120
14.5	2.2549599332	2.2549599332
14.6	2.2571748593	2.2571748593
14.7	2.2591855886	2.2591855887
14.8	2.2609888515	2.2609888516
14.9	2.2625817157	2.2625817158
15.0	2.2639615911	2.2639615912
15.1	2.2651262338	2.2651262339
15.2	2.2660737500	2.2660737501
15.3	2.2668025990	2.2668025991
15.4	2.2673115956	2.2673115957
15.5	2.2675999121	2.2675999122

15.6	2.2676670797	2.2676670798
15.7	2.2675129892	2.2675129892
15.8	2.2671378911	2.2671378911
15.9	2.2665423953	2.2665423954
16.0	2.2657274703	2.2657274703
16.1	2.2646944411	2.2646944412
16.2	2.2634449876	2.2634449876
16.3	2.2619811415	2.2619811415
16.4	2.2603052831	2.2603052831
16.5	2.2584201375	2.2584201376
16.6	2.2563287703	2.2563287703
16.7	2.2540345821	2.2540345821
16.8	2.2515413035	2.2515413035
16.9	2.2488529888	2.2488529888
17.0	2.2459740095	2.2459740095
17.1	2.2429090471	2.2429090471
17.2	2.2396630854	2.2396630855
17.3	2.2362414029	2.2362414029
17.4	2.2326495633	2.2326495634
17.5	2.2288934075	2.2288934076
17.6	2.2249790432	2.2249790433
17.7	2.2209128357	2.2209128358
17.8	2.2167013969	2.2167013970
17.9	2.2123515751	2.2123515751
18.0	2.2078704434	2.2078704435
18.1	2.2032652887	2.2032652888
18.2	2.1985435993	2.1985435994
18.3	2.1937130533	2.1937130533
18.4	2.1887815054	2.1887815055
18.5	2.1837569749	2.1837569750
18.6	2.1786476322	2.1786476322
18.7	2.1734617855	2.1734617855
18.8	2.1682078675	2.1682078675
18.9	2.1628944216	2.1628944216
19.0	2.1575300880	2.1575300880
19.1	2.1521235895	2.1521235896
19.2	2.1466837178	2.1466837178
19.3	2.1412193184	2.1412193185
19.4	2.1357392771	2.1357392772
19.5	2.1302525050	2.1302525051
19.6	2.1247679240	2.1247679241
19.7	2.1192944526	2.1192944527
19.8	2.1138409913	2.1138409913
19.9	2.1084164078	2.1084164078
20.0	2.1030295230	2.1030295230
20.1	2.0976890966	2.0976890966
20.2	2.0924038125	2.0924038126
20.3	2.0871822652	2.0871822653
20.4	2.0820329455	2.0820329455
20.5	2.0769642265	2.0769642265
20.6	2.0719843505	2.0719843505
20.7	2.0671014153	2.0671014153
20.8	2.0623233610	2.0623233610

20.9	2.0576579571	2.0576579572
21.0	2.0531127901	2.0531127902
21.1	2.0486952509	2.0486952509
21.2	2.0444125227	2.0444125228
21.3	2.0402715698	2.0402715698
21.4	2.0362791256	2.0362791257
21.5	2.0324416824	2.0324416824
21.6	2.0287654800	2.0287654801
21.7	2.0252564965	2.0252564966
21.8	2.0219204378	2.0219204378
21.9	2.0187627285	2.0187627286
22.0	2.0157885035	2.0157885035
22.1	2.0130025991	2.0130025991
22.2	2.0104095454	2.0104095455
22.3	2.0080135591	2.0080135591
22.4	2.0058185362	2.0058185362
22.5	2.0038280460	2.0038280460
22.6	2.0020453253	2.0020453253
22.7	2.0004732729	2.0004732730
22.8	1.9991144452	1.9991144452
22.9	1.9979710517	1.9979710518
23.0	1.9970449517	1.9970449518
23.1	1.9963376512	1.9963376512
23.2	1.9958503002	1.9958503002
23.3	1.9955836912	1.9955836913
23.4	1.9955382579	1.9955382579
23.5	1.9957140740	1.9957140740
23.6	1.9961108536	1.9961108537
23.7	1.9967279516	1.9967279517
23.8	1.9975643646	1.9975643646
23.9	1.9986187323	1.9986187323
24.0	1.9998893403	1.9998893404
24.1	2.0013741226	2.0013741226
24.2	2.0030706646	2.0030706646
24.3	2.0049762076	2.0049762077
24.4	2.0070876531	2.0070876531
24.5	2.0094015676	2.0094015677
24.6	2.0119141885	2.0119141886
24.7	2.0146214300	2.0146214301
24.8	2.0175188900	2.0175188900
24.9	2.0206018567	2.0206018568
25.0	2.0238653171	2.0238653172
25.1	2.0273039644	2.0273039645
25.2	2.0309122071	2.0309122072
25.3	2.0346841779	2.0346841779
25.4	2.0386137430	2.0386137431
25.5	2.0426945128	2.0426945128
25.6	2.0469198513	2.0469198514
25.7	2.0512828880	2.0512828880
25.8	2.0557765280	2.0557765280
25.9	2.0603934642	2.0603934643
26.0	2.0651261891	2.0651261892
26.1	2.0699670068	2.0699670068

26.2	2.0749080457	2.0749080457
26.3	2.0799412711	2.0799412711
26.4	2.0850584986	2.0850584986
26.5	2.0902514070	2.0902514070
26.6	2.0955115522	2.0955115522
26.7	2.1008303807	2.1008303807
26.8	2.1061992435	2.1061992436
26.9	2.1116094104	2.1116094105
27.0	2.1170520840	2.1170520840
27.1	2.1225184138	2.1225184138
27.2	2.1279995112	2.1279995112
27.3	2.1334864633	2.1334864633
27.4	2.1389703478	2.1389703479
27.5	2.1444422475	2.1444422475
27.6	2.1498932644	2.1498932644
27.7	2.1553145347	2.1553145347
27.8	2.1606972429	2.1606972429
27.9	2.1660326362	2.1660326362
28.0	2.1713120387	2.1713120388
28.1	2.1765268656	2.1765268657
28.2	2.1816686372	2.1816686372
28.3	2.1867289923	2.1867289923
28.4	2.1916997024	2.1916997024
28.5	2.1965726846	2.1965726846
28.6	2.2013400150	2.2013400150
28.7	2.2059939414	2.2059939414
28.8	2.2105268961	2.2105268961
28.9	2.2149315081	2.2149315082
29.0	2.2192006151	2.2192006151
29.1	2.2233272751	2.2233272751
29.2	2.2273047777	2.2273047777
29.3	2.2311266551	2.2311266552
29.4	2.2347866927	2.2347866927
29.5	2.2382789387	2.2382789388
29.6	2.2415977146	2.2415977147
29.7	2.2447376237	2.2447376237
29.8	2.2476935601	2.2476935601
29.9	2.2504607172	2.2504607172
30.0	2.2530345954	2.2530345954
30.1	2.2554110092	2.2554110093
30.2	2.2575860945	2.2575860946
30.3	2.2595563143	2.2595563144
30.4	2.2613184648	2.2613184649
30.5	2.2628696807	2.2628696808
30.6	2.2642074395	2.2642074395
30.7	2.2653295658	2.2653295658
30.8	2.2662342350	2.2662342350
30.9	2.2669199760	2.2669199761
31.0	2.2673856737	2.2673856738
31.1	2.2676305709	2.2676305710
31.2	2.2676542694	2.2676542694
31.3	2.2674567305	2.2674567305
31.4	2.2670382755	2.2670382756

31.5	2.2663995849	2.2663995850
31.6	2.2655416972	2.2655416973
31.7	2.2644660075	2.2644660075
31.8	2.2631742649	2.2631742649
31.9	2.2616685698	2.2616685699
32.0	2.2599513708	2.2599513709
32.1	2.2580254602	2.2580254602
32.2	2.2558939696	2.2558939697
32.3	2.2535603651	2.2535603651
32.4	2.2510284413	2.2510284413
32.5	2.2483023154	2.2483023154
32.6	2.2453864203	2.2453864204
32.7	2.2422854976	2.2422854977
32.8	2.2390045896	2.2390045897
32.9	2.2355490315	2.2355490316
33.0	2.2319244423	2.2319244423
33.1	2.2281367158	2.2281367159
33.2	2.2241920115	2.2241920115
33.3	2.2200967436	2.2200967436
33.4	2.2158575715	2.2158575715
33.5	2.2114813885	2.2114813885
33.6	2.2069753107	2.2069753107
33.7	2.2023466654	2.2023466654
33.8	2.1976029792	2.1976029792
33.9	2.1927519658	2.1927519659
34.0	2.1878015135	2.1878015136
34.1	2.1827596721	2.1827596722
34.2	2.1776346402	2.1776346403
34.3	2.1724347516	2.1724347516
34.4	2.1671684617	2.1671684617
34.5	2.1618443341	2.1618443341
34.6	2.1564710262	2.1564710263
34.7	2.1510572757	2.1510572758
34.8	2.1456118859	2.1456118859
34.9	2.1401437113	2.1401437113
35.0	2.1346616438	2.1346616439
35.1	2.1291745979	2.1291745979
35.2	2.1236914958	2.1236914959
35.3	2.1182212537	2.1182212538
35.4	2.1127727668	2.1127727669
35.5	2.1073548948	2.1073548948
35.6	2.1019764476	2.1019764476
35.7	2.0966461711	2.0966461712
35.8	2.0913727330	2.0913727330
35.9	2.0861647082	2.0861647083
36.0	2.0810305656	2.0810305657
36.1	2.0759786538	2.0759786538
36.2	2.0710171876	2.0710171876
36.3	2.0661542348	2.0661542349
36.4	2.0613977032	2.0613977033
36.5	2.0567553273	2.0567553273
36.6	2.0522346559	2.0522346560
36.7	2.0478430403	2.0478430403

36.8	2.0435876214	2.0435876215
36.9	2.0394753192	2.0394753193
37.0	2.0355128206	2.0355128206
37.1	2.0317065689	2.0317065690
37.2	2.0280627535	2.0280627536
37.3	2.0245872997	2.0245872997
37.4	2.0212858588	2.0212858588
37.5	2.0181637992	2.0181637993
37.6	2.0152261978	2.0152261979
37.7	2.0124778314	2.0124778315
37.8	2.0099231691	2.0099231691
37.9	2.0075663650	2.0075663650
38.0	2.0054112515	2.0054112515
38.1	2.0034613330	2.0034613330
38.2	2.0017197803	2.0017197803
38.3	2.0001894252	2.0001894253
38.4	1.9988727565	1.9988727565
38.5	1.9977719149	1.9977719149
38.6	1.9968886907	1.9968886907
38.7	1.9962245199	1.9962245200
38.8	1.9957804828	1.9957804828
38.9	1.9955573012	1.9955573012
39.0	1.9955553380	1.9955553381
39.1	1.9957745966	1.9957745967
39.2	1.9962147203	1.9962147204
39.3	1.9968749935	1.9968749936
39.4	1.9977543425	1.9977543425
39.5	1.9988513374	1.9988513374
39.6	2.0001641943	2.0001641944
39.7	2.0016907785	2.0016907785
39.8	2.0034286075	2.0034286076
39.9	2.0053748556	2.0053748556
40.0	2.0075263578	2.0075263579

Column 1 shows the elapsed simulation time in fs. Columns 2-3 show the calculated carbon-oxygen bond lengths (in a_0) predicted by the Solidity and C# implementations of the trajectory, respectively.
